# Kinetics of cone specific G-protein signaling in avian photoreceptor cells

**DOI:** 10.3389/fnmol.2023.1107025

**Published:** 2023-01-17

**Authors:** Chad Yee, Katharina Görtemaker, Rieke Wellpott, Karl-Wilhelm Koch

**Affiliations:** ^1^Division of Biochemistry, Department of Neuroscience, University of Oldenburg, Oldenburg, Germany; ^2^Research Center Neurosensory Sciences, University of Oldenburg, Oldenburg, Germany

**Keywords:** photoreceptor, G protein, cone outer segment, cone opsin, protein–protein interaction

## Abstract

Cone photoreceptor cells of night-migratory songbirds seem to process the primary steps of two different senses, vision and magnetoreception. The molecular basis of phototransduction is a prototypical G protein-coupled receptor pathway starting with the photoexcitation of rhodopsin or cone opsin thereby activating a heterotrimeric G protein named transducin. This interaction is well understood in vertebrate rod cells, but parameter describing protein–protein interactions of cone specific proteins are rare and not available for migratory birds. European robin is a model organism for studying the orientation of birds in the earth magnetic field. Recent findings showed a link between the putative magnetoreceptor cryptochrome 4a and the cone specific G-protein of European robin. In the present work, we investigated the interaction of European robin cone specific G protein and cytoplasmic regions of long wavelength opsin. We identified the second loop in opsin connecting transmembrane regions three and four as a critical binding interface. Surface plasmon resonance studies using a synthetic peptide representing the second cytoplasmic loop and purified G protein *α*-subunit showed a high affinity interaction with a *K*_D_ value of 21 nM. Truncation of the G protein *α*-subunit at the C-terminus by six amino acids slightly decreased the affinity. Our results suggest that binding of the G protein to cryptochrome can compete with the interaction of G protein and non-photoexcited long wavelength opsin. Thus, the parallel presence of two different sensory pathways in bird cone photoreceptors is reasonable under dark-adapted conditions or during illumination with short wavelengths.

## Introduction

Vertebrate phototransduction is a sensory signaling pathway providing the link between a physical stimulus (photon) and a change in membrane potential. The molecular reactions of the biochemical cascade involving the prototypical G protein-coupled receptors (GPCR) rhodopsin or cone opsin, a heterotrimeric G protein (transducin, G_t_), a cGMP-specific phosphodiesterase (PDE), and a cyclic nucleotide-gated cation channel (CNG-channel) that is directly controlled by the intracellular cGMP concentration, are understood in quantitative terms (Pugh and Lamb, 2000; Chen et al., 2022; Hofmann and Lamb, 2022). Similar holds true for the deactivation steps of each of the biochemical reactions in the photoexcitation process. Regulatory feedback mechanisms further control the recovery of the cell to the dark or light adapted state (Koch and Dell’Orco, 2015; Dizhoor and Peshenko, 2021). The principal signaling components are present in rod and cone photoreceptor cells, which however, express rod or cone specific isoforms of these components. Differences in light sensitivity and photoresponse kinetics probably originate from different biochemical properties of rod or cone specific protein isoforms, but quantitative parameters derived from cone specific proteins are less available than those obtained with rod specific proteins (Mustafi et al., 2009; Fain and Sampath, 2021; Kawamura and Tachibanaki, 2022). Biochemical and biophysical parameters based on experiments stimulated various computer assisted mathematical modeling approaches of the vertebrate photoresponse in rod outer segments (Hamer et al., 2003; Dell’Orco et al., 2009; Invergo et al., 2013, 2014; Beelen et al., 2021). Similar models of cone phototransduction are very limited so far, and recent attempts focus on the different morphology of rod and cone outer segments (Klaus et al., 2021).

Most information of phototransduction has been obtained from studies on mammalian, amphibian, and zebrafish. In contrast, very limited research was performed on the bird retina focusing primarily on cone visual pigments and the use of chicken eye as a research model for ocular diseases (Wisely et al., 2017). Very recently, however, the magnetic sense of night-migratory songbirds is the second sense, beside vision, that is associated with processes in the retina. Cryptochrome (Cry) flavoproteins are currently discussed as the primary sensing molecule mediating a radical-pair mechanism (Schulten et al., 1978; Wiltschko et al., 1993; Ritz et al., 2000; Hore and Mouritsen, 2016) and different Cry isoforms are expressed in different layers of bird species (Liedvogel et al., 2007; Niessner et al., 2011, 2016; Günther et al., 2018; Bolte et al., 2021). Xu et al. (2021) demonstrated magnetic sensitivity of photo-induced radical pair formation in European robin Cry4a making this cryptochrome variant a prime magnetoreceptor candidate. In a separate study, Hochstoeger et al. (2020) presented evidence for pigeon Cry4 acting as an ultraviolet-blue photoreceptor that forms photo-induced radical pairs. A previous study reported that Cry4a directly interacts with the α–subunit of a cone specific heterotrimeric G protein (Gtα) from European robin (*Erithacus rubecula*) (Wu et al., 2020). The G protein Gtα is among a group of six proteins that were identified in a previous yeast-two-hybrid screening as putative Cry4 binding partners of the European robin. In addition to Gtα, the group consists of the γ-subunit of the cone specific heterotrimeric G-protein (Gtγ), long-wavelength-sensitive opsin (LWO), the subunit Kv8.2 of the voltage-gated heteromeric potassium channel Kv2/Kv8.2, the retinol binding protein 1 and retinal G protein-coupled receptor (Wu et al., 2020). Görtemaker et al. (2022) verified the interaction of Cry4a and Gtα by surface plasmon resonance (SPR) spectroscopy, biochemical pulldown tests, and Förster resonance energy transfer measurements.

Any downstream steps triggered by the Cry4a-Gtα interaction are unknown so far and the identification of LWO as one of the putative Cry4a binding partner raises additional questions about a possible interference between phototransduction and magnetoreception in bird cone outer segments. Current hypothetical models discuss Cry4a formation with Gtα or LWO as being part of the classical phototransduction cascade or as a starting point of an unknown pathway (Wu et al., 2020). The SPR study by Görtemaker et al. (2022) employed purified Cry4a and Gtα that interacted with high to moderate affinity yielding dissociation constants (*K*_D_) in the lower nanomolar range. However, the process of Gtα interacting with European robin LWO has not been investigated so far and therefore affinity constants of the binding process are unknown. It is of crucial importance to derive a quantitative understanding of Gtα coupling to LWO, not only to complement mathematical models of phototransduction, but also to compare phototransduction with processes involved in bird magnetoreception. In the current contribution, we asked which of the hypothetical cytoplasmic loops of LWO interact with Gtα and what the binding affinities are. We used SPR to determine affinity constants and confirmed the binding process by a fluorescence-based interaction assay.

## Materials and methods

### Peptides

**Figure 1:**
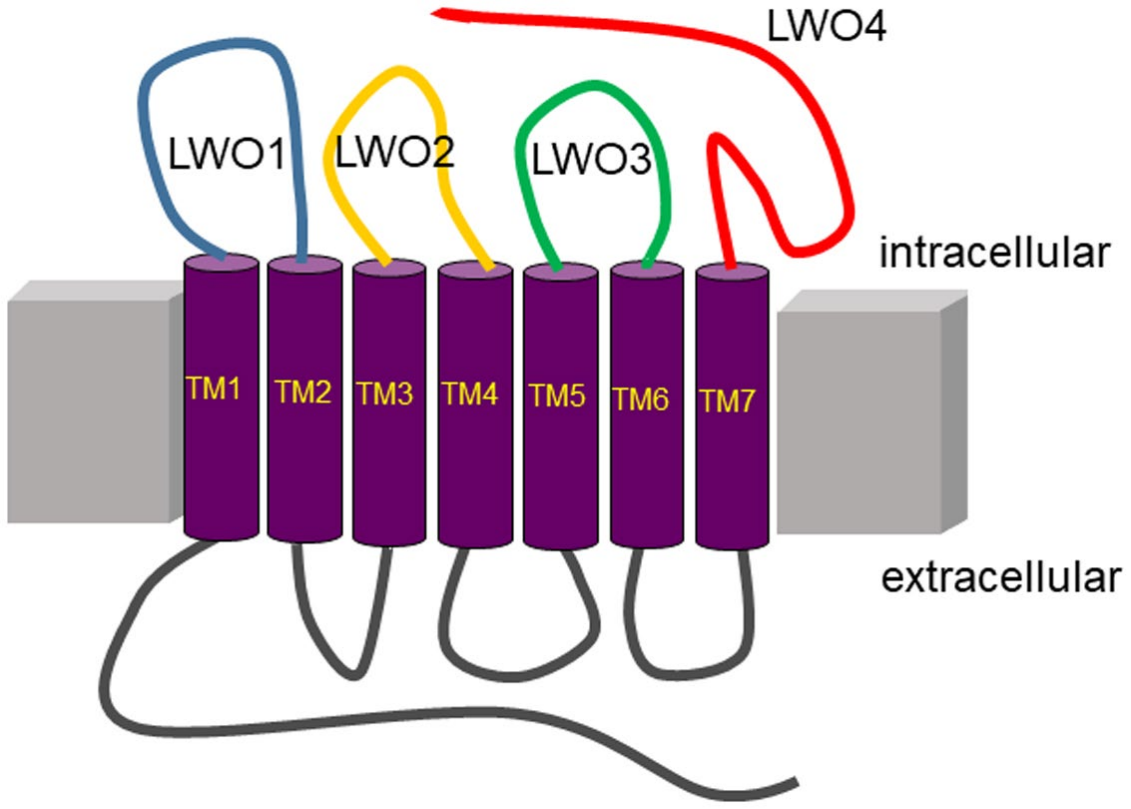
Topography of long wavelength opsin from European robin. Peptides used in the present study are on the intracellular (cytoplasmic) side of the membrane protein indicated by the colored loops (blue, LWO1; yellow, LWO2; green, LWO3; red, LWO4). Extracellular loops are in grey. The seven transmembrane regions (TM1–TM7) are connected *via* the loops and are presented in purple.

The peptides representing the cytoplasmic loops of LWO (Figure 1) were purchased from Genscript (Netherlands). Sequences of the peptides were deduced from genetic sequences of the European robin genome (Dunn et al., 2021). Two control peptides were ordered containing scrambled sequences of LWO2 and LWO3 assigned as LWO2-sc and LWO3-sc, respectively (Table 1). To the N-terminus of each peptide, a linker (CGAGA or CGAGAG) was added to allow for specific covalent coupling *via* cysteine. All peptides were ordered to a purity of >90% and contained no further N-terminal modifications. The C-terminus of peptides LWO-1-3 were amidated to simulate a continuation of the peptide chain, while LWO-4 was left unmodified to represent the opsin C-terminus. Quality control by LC–MS was performed by Genscript, and concentrations of the peptides were confirmed by UV–vis measurements.

**Table 1:** Amino acid sequences of the LWO peptides used in the present investigation.

Peptide name	Amino acid sequence
LWO1	CGAGA-TAKFKKLRHPLNWI
LWO2	CGAGA-WERWFVVCKPFGNIKFDGK
LWO3	CGAGAG-AIRAVAAQQKESESTQKAEKEVSR
LWO4	CGAGAG-NRQFRNCILQLFGKKVDDGSEVSTSRTEVSSVSNSSGS
LWO2-sc (scrambled)	CGAGA-FRVKDGWINPGKFEVWCKF
LWO3-sc (scrambled)	CGAGA-GSKSQRARAEQVETAQAKAEISVEK

N-terminal sequences CGAGA and CGAGAG are linker sequence additions. The sequence of the LWO was based on genome sequence information of European robin published in Dunn et al. (2021). The second last amino acid in LWO4 is G instead of V due to preliminary sequence information at the time of peptide synthesis.

### Cloning and expression of Gtα variants

We used a Gtα/Giα chimera to allow functional expression in *E. coli* according to previous attempts with bovine Gtα (Skiba et al., 1996). Cloning of the Gtα variants was performed by PCR mutagenesis of the plasmid described in Görtemaker et al. (2022). In short, the construct contained an N-terminal 6x histidine tag and SUMO (Small Ubiquitin like MOdifier) protein (for metal affinity purification and subsequent tag cleavage) followed by the Gtα chimera. Truncation of 3 and 6 amino acids was performed using the primers 1 and 2 and 1 and 3, respectively. Sequences are available in Supplementary Table S1. The truncated variants were expressed following the same protocol as the full-length protein.

### Purification of proteins and protein analysis

Purification of the Gtα/Giα chimera was performed as exactly described previously (Görtemaker et al., 2022). We used the same procedure for purification of the truncated Gtα variants, but we excluded the final size exclusion chromatography. Protein samples were analyzed by standard analytical techniques such as sodium dodecyl-sulfate polyacrylamide gel electrophoresis (SDS-PAGE) and protein quantification according to established procedures in the laboratory (Elbers et al., 2018).

### Functional test of Gtα variants

We tested the functional status of purified chimeric Gtα variants by intrinsic Trp fluorescence. G protein α-subunits of heterotrimeric G proteins harbor a conserved Trp residue at or near position 207 that monitors an activation-dependent conformational change in α-subunits triggered by the binding of Mg^2+^-GDP and AlF4-resembling the transition to the active state (Faurobert et al., 1993; Mazzoni and Hamm, 1993; Preininger et al., 2008; Hamm et al., 2009). We recorded relative fluorescence emission using purified G_t_α/Giα variants and a spectrofluorimeter from Photon Technology International exactly as described recently by us (Görtemaker et al., 2022).

### Surface plasmon resonance

Surface plasmon resonance (SPR) measurements were performed on a Biacore 3,000 (GE Healthcare now Cytiva). We followed the general operation principle that had been described before (Koch, 2000; Komolov et al., 2009). In the present work, we immobilized the non-myristoylated Gtα/Giα chimera or its truncated variants using CM5 sensor chips (GE Healthcare) for all applications exactly as previously described (Görtemaker et al., 2022). Immobilization densities of Gtα/Giα were 2.6–3.4 ng/mm^2^. The truncated variants Gt-3AA and Gt-6AA bound to the senor chip surface at densities of 3.1–4.2 ng/mm^2^. First, we studied the interaction processes by injection of peptides representing the cytoplasmic regions in LWO (Table 1). Two peptides made from the amino acids present in LWO2 and LWO3, but in scrambled order (Table 1), served as controls. We injected different concentrations (10, 50, 100, and 200 nM or alternatively: 10, 25, 45, 55, 75, and 100 nM) at a flow rate of either 25 μl/min or 50 μl/min in SPR running buffer (10 mM HEPES/NaOH, pH 7.4, 150 mM NaCl, 10 mM MgCl_2_, 0.005% Tween-20, 3.4 mM EDTA). In addition, we coated control surfaces with ubiquitin-like-protease 1 as previously described (Görtemaker et al., 2022). For kinetic investigation of LWO2 binding to Gtα/Giα, we injected different peptide concentrations in random order. Regeneration of the surface was performed with a basic-detergent cocktail adopted from Andersson et al. (1999) containing final concentrations of 12.5 mM of ethanolamine, Na_2_PO_4_, piperazine, and glycine set to a pH of 11.75, as well as containing 0.2% sodium dodecyl sulfate. Despite the relatively harsh conditions, regeneration was judged to be slightly incomplete. Increasing the pH or SDS concentration did not improve the results, and other tested conditions were unsatisfactory. With the conditions used, reproducible data was still obtained. Sensorgrams were evaluated by nonlinear curve fitting applying the global fitting approach (BIAevaluation software 4.1, GE Healthcare). Association and dissociation rate constants (*k*_a_ and *k*_d_, respectively) yielded apparent *K*_D_ values from the ratio of *k*_d_/*k*_a_. Data derived from immobilized Gtα/Giα were obtained from 12 different sets. Data derived from truncated Gtα/Giα were obtained from three different sets.

### Förster resonance energy transfer

Interaction of the Gtα/Giα chimera with peptide LWO2 and the control peptide LWO2-sc was tested by Förster resonance energy transfer (FRET) measurements. We designed an experiment using fluorescence excitation at 280 nm of endogenous Trp residues in Gtα/Giα and peptide and emission of Trp fluorescence at 334 nm that excited the fluorescence dye 5-Dimethylamino-1-naphthalinsulfonyl chloride (dansyl chloride). The emission spectrum of dansyl chloride was recorded between 400 and 550 nm. For this purpose, we coupled dansyl chloride to free amino groups in LWO2 and LWO2-sc. Peptides were solved in H_2_0 bidest (1 mg/ml) and 66 μl of a peptide solution were mixed with 50 μl borat buffer (0.1 M, pH 9.5). Twenty-five μL dansyl chloride (1 mg/ml in acetone) were added and the solutions were incubated at room temperature for 5 h in darkness. Afterwards, we separated non-reacted dansyl chloride, non-reacted peptides and covalently labeled LWO2 or LWO2-sc by reversed phase liquid chromatography (HPLC) using a LiChrospher® 100 RP-18 (5 μm) column (Merck, Germany) in a Hitachi Primaide HPLC system. The column was equilibrated in 0.1% trifluoro-acetic acid (v/v) in H_2_O bidest. Separation was achieved by applying a gradient from 0.1% trifluoro-acetic acid (v/v) in H_2_O bidest to 100% acetonitrile with 0.1% trifluoro-acetic acid (v/v) in 55 min. Peaks were detected at 280 nm and the area was used to calculate the coupling yield that was at 84–97% for both peptides. FRET measurements employing the FRET pair intrinsic tryptophan and attached dansyl were performed with a fluorescence spectrometer from photon Technology International. The basic operation principle was essentially as described before (Behnen et al., 2009; Scholten and Koch, 2011) using the following modifications. Shortly before recording the spectrum, the dansylated LWO2 (or LWO2-sc) containing fraction of the HPLC elution was diluted in fluorescence buffer (80 mM Hepes pH 7.4, 40 mM KCl, 150 mM NaCl, 10 mM MgCl_2_) at a final concentration of 16.7 μM. After adding the G protein, the mixtures were incubated on ice in the dark for 30 min. During the measurements with the Gtα/Giα, it was present in GDP-bound conformation ([GDP] = 10 μM). The excitation wavelength was set to 280 nm and the emission spectrum was recorded from 400 to 550 nm. Recording and analysis of the data was performed using Photon Technology International software package FELIX32.

## Results

### Identification of Gtα binding sites in European robin long-wavelength-sensitive opsin

**Figure 2:**
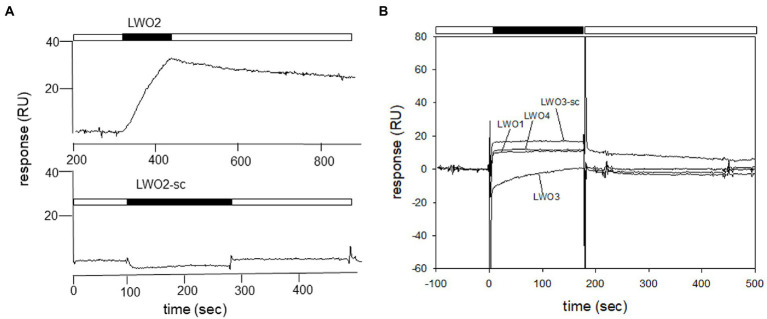
Identification of Gtα/Giα interacting cytoplasmic loops by SPR. Purified Gtα/Giα was immobilized on a CM5 sensor chip *via* amine coupling. Peptides were dissolved in SPR running buffer and flushed over the surface at a concentration of 100 nM. **(A)** Comparison of sensorgrams recorded with LWO2 and the control peptide LWO2-sc. The sensorgram of the LWO2 experiment (upper trace) starts at 200 s, because the recording had a longer buffer run before injection than the experiment with LWO2-sc (lower trace) and we cut the prerun containing no information. The black bars indicate the injection of the peptides, white bars show flowing of running buffer. When the injection of the peptide stops, the flow of running buffer triggers the dissociation of the LWO2-Gtα/Giα complex. **(B)** Sensorgrams displaying the injection of peptides LWO1, LWO3, LWO4, and LWO3-sc. Black and white bars as in **(A)**.

Since LWO from European robin is a prototypical GPCR, its cytoplasmic regions connecting the transmembrane region or extending from transmembrane helix VII provide possible interaction sites for cytoplasmic signaling proteins (Figure 1). We used custom-made peptides representing all four cytoplasmic regions (Figure 1 and Table 1) of LWO in a screening test. The interacting Gtα was expressed and purified as a Gtα/Giα chimera, which is a common way to obtain photoreceptor specific Gtα proteins in soluble and active form (Skiba et al., 1996). We tested the function of the Gtα/Giα by a tryptophan fluorescence assay that monitors the transition of GDP-bound Gtα/Giα from an inactive to the active state triggered by the addition of AlF4-exactly as performed and described (Görtemaker et al., 2022). All Gtα/Giα variants proved to be active and were suitable for interaction studies. The Gtα/Giα chimera was the immobilized ligand in the SPR experiments following a similar design like in our previous study about Gtα/Giα and Cry4a complex formation (Görtemaker et al., 2022). Injection of LWO2 resulted in a positive resonance signal exhibiting Langmuir binding kinetics representing a 1:1 complex formation (upper trace in Figure 2A, see below for a more detailed analysis). Injection of the control peptide LWO2-sc caused slight decrease in resonance units indicating no binding signal (lower trace in Figure 2A). Peptides LWO1, LWO4 and LWO3-sc caused a positive rectangular response between 10 and 20 RU (Figure 2B) that is typical for changes in bulk refractive index and might originate from a minor mismatch in buffer composition. Therefore, it did not indicate a binding process. Peptide LWO3 showed a different behavior, since we observed a slight decrease in RU and a kind of recovery phase (Figure 2B) reaching the zero baseline at the end of injection. During subsequent buffer flow, a positive response of *ca.* 10 RU maintained, but decreased over time. Although the shape of the response curve was different from that obtained with LWO2, it could have indicated a binding process of lower affinity. We therefore doubled the concentration of LWO3 during injection, but recorded only a negative rectangular response of ~ −10 RU showing that no specific binding process occurred (Supplementary Figure S1).

### Kinetic analysis of Gtα/Giα interacting with LWO2 peptide

**Figure 3:**
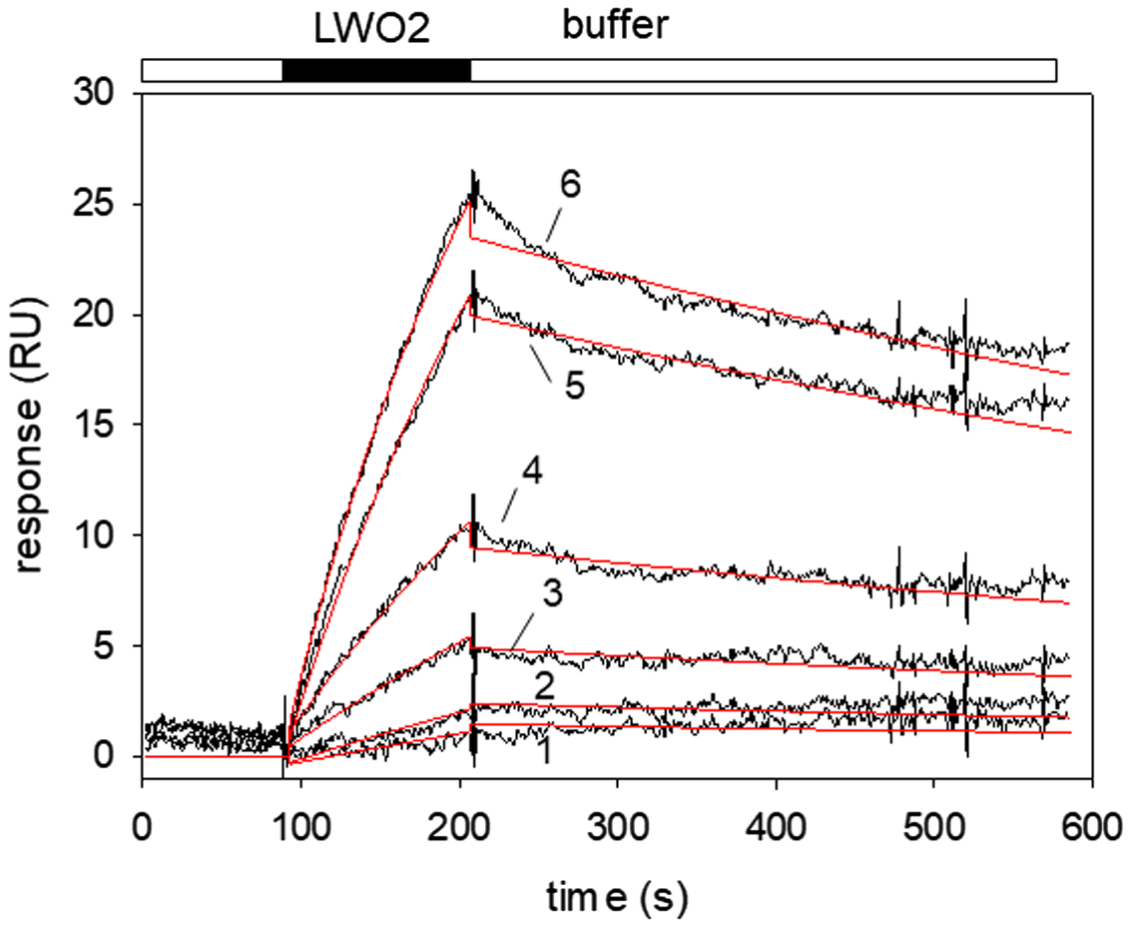
Surface plasmon resonance (SPR) recordings of LWO2 interacting with immobilized Gtα/Giα. Black bar indicates the injection of different peptide concentrations resulting in the association phase, the open bar indicates buffer flow and the dissociation phase, when the injection of the peptide stops. Larger RU values resulted from the injection of higher LWO2 concentrations. Sensorgrams (black lines) obtained after flushing of 5 nM (1), 15 nM (2), 30 nM (3), 50 nM (4), 75 nM (5), and 100 nM (6) LWO2 over immobilized Gtα/Giα lead to the formation of a LWO2-Gtα/Giα complex. Global curve fitting (Langmuir 1:1 binding model, red lines) resulted in an association rate constant *k*_a_ = 5.36 × 10^4^ M^−1^ s^−1^ and a dissociation rate constant *k*_d_ = 8.12 × 10^−4^ s^−1^, *K*_D_ = 15.1 nM. The set of sensorgrams is representative of 12 different sets (see main text for mean *K*_D_).

We continued with a more extensive kinetic study of LWO2 binding to Gtα/Giα and injected different concentrations of LWO2 onto a Gtα/Giα coated sensor chip surface (Figure 3). We varied the LWO2 concentration between 5 and 150 nM in different sets of sensorgrams similar to the one shown in Figure 3. We applied a simple Langmuir binding model (A + B ↔ AB) for nonlinear curve fitting using the global fitting option of the BIAevaluation software. For example, fitting the sensorgrams (recording in black, fits in red) in Figure 3 gave an association rate constant of 5.36 × 10^4^ M^−1^ s^−1^ and a dissociation rate constant of 8.12 × 10^−4^ s^−1^ resulting in a K_D_ of 15.1 nM. Evaluation of 12 different sets resulted in a *K*_D_ = 21.4 nM ± 14.5 nM. We observed a large variation in the *K*_D_ values obtained from different sets spanning the range from 3 to 41 nM.

### Gtα/Giα interacting with LWO2 peptide in solution

**Figure 4:**
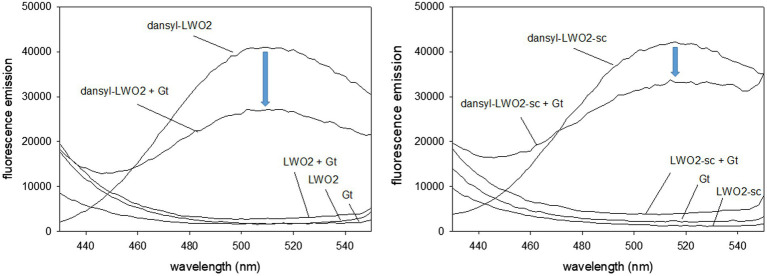
Fluorescence study with dansylated peptides dansyl-LWO2 and dansyl-LWO2-sc. Peptides and Gtα/Giα were present at equal concentration of 16.7 μM. Excitation was at 280 nm, the emission was recorded from 430 to 550 nm. Left panel: FRET measurements of dansyl-LWO2 in the absence and presence of Gtα/Giα (indicated as Gt); right panel: FRET measurements of dansyl-LWO2-sc in the absence and presence of Gtα/Giα. No emission was observed with label-free peptides in the absence and presence of Gtα/Giα.

We further tested the interaction of LWO2 with Gtα/Giα in solution using FRET measurements. Emission of intrinsic Trp in Gtα/Giα and LWO2 (or LWO2-sc) excited the dansyl dye covalently bound to LWO2 (or LWO2-sc). Dansyl-LWO2 showed a high fluorescence emission with a maximum at 510 nm that decreased in the presence of Gtα/Giα by 14,000 relative fluorescence counts (Figure 4, left panel). No fluorescence emission was observed, when LWO2, Gtα/Giα, and LWO2 + Gtα/Giα were separately tested (Figure 4, left panel). The scrambled peptide dansyl-LWO2-sc showed a similar high fluorescence emission with a λ_max_ at 516 nm. The shift might come from the different Trp positions in the scrambled version of LWO2. Addition of Gtα/Giα decreased the relative fluorescence counts, but the decrease was less reaching only 9,000 counts (Figure 4, right panel). No fluorescence emission was also observed with LWO2-sc, Gtα/Giα, and LWO2-sc + Gtα/Giα. We conclude from comparing the decrease in fluorescence emission between both experiments (see blue arrow) that Gtα/Giα interferes more with the FRET signal of dansyl-LWO2 than with those from dansyl-LWO2-sc indicating an interaction of Gtα/Giα and LWO2 in solution. The high background seen with the scrambled LWO2 peptide seems to be caused by the dansyl moiety interacting non-specifically with Gtα/Giα. However, this is clearly less than the interference of Gtα/Giα with the FRET measured with dansyl-LWO2.

### Truncation of Gtα/Giα at the C-terminus

**Figure 5:**
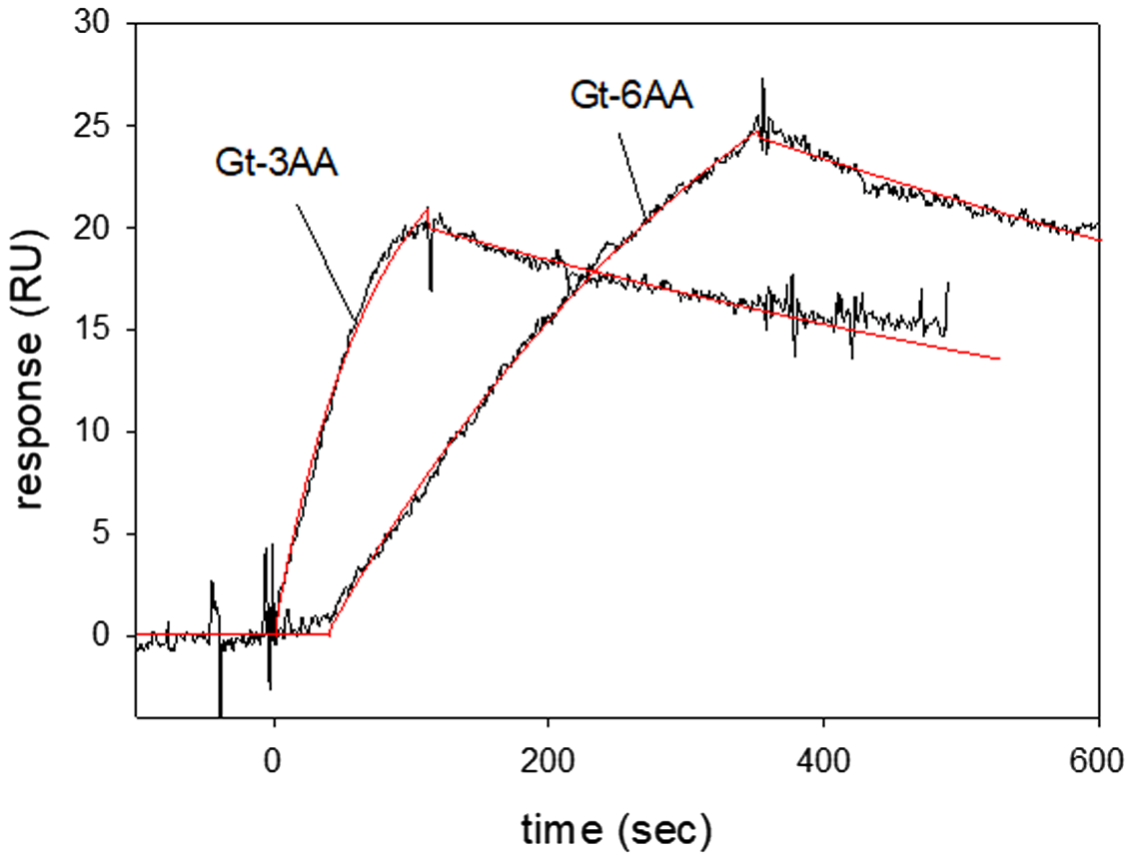
Surface plasmon resonance recordings of LWO2 interacting with truncated variants of Gtα/Giα. Gtα/Giα with a C-terminus truncated by three or six amino acids (Gt-3AA and Gt-6AA, respectively) was immobilized on a CM5 sensor chip. Peptide LWO2 was injected and flushed over the surface at a concentration of 120 nM (Gt-3AA) or 100 nM (Gt-6AA). Global curve fitting (Langmuir 1:1 binding model, red lines) resulted for Gt-3AA in an association rate constant *k*_a_ = 1.08 × 10^5^ M^−1^ s^−1^ and a dissociation rate constant *k*_d_ = 9.37 × 10^−4^ s^−1^, *K*_D_ = 8.7 nM. For Gt-6AA we obtained an association rate constant *k*_a_ = 1.86 × 10^4^ M^−1^ s^−1^ and a dissociation rate constant *k*_d_ = 9.3 × 10^−4^ s^−1^, *K*_D_ = 50 nM. The sensorgrams are representative of three different sets for each Gtα/Giα variant.

The extreme C-terminus of the α-subunit in bovine transducin interacts with rhodopsin during light activation (Herrmann et al., 2006; Scheerer et al., 2008). We systematically truncated the C-terminus of Gtα/Giα by three and six amino acids and tested its binding capability to LWO-2. All purified truncated Gtα/Giα variants were functional as tested by AlF_4_^−^/Trp fluorescence assay (Supplementary Figure S2). SPR interaction studies using immobilized truncated Gtα/Giα variants showed interaction with LWO-2. Removing three amino acids (Gt-3AA) did not influence the binding kinetics, since we observed similar association and dissociation rate constants and a *K*_D_-value in the lower nanomolar range (Figure 5). Three different sets injecting 120 nM LWO2 resulted in a *K*_D_ = 7.3 nM ± 1.2 nM, which was in the range that we observed with non-truncated Gtα/Giα. Interaction of 100 nM LWO2 with Gt-6AA yielded a *K*_D_ = 34.5 nM ± 13.8 nM. Thus, the affinity decreased by a factor 1.6 (34.5/21.4), when compared with the non-truncated variant. This decrease was mainly caused by the lower association rate constant as shown in Figure 5. When we increased the concentration of the peptide, we recorded larger RU values, but the binding kinetics did not obey to a simple Langmuir binding model (1:1). Instead, satisfying fits using the global fit approach were only obtained with additional assumptions concerning the binding process on the sensor chip surface (for example, complex formation with a sequential conformational change, see Supplementary Figure S3).

## Discussion

Sensory cells receive physical or chemical stimuli that trigger specific biochemical signaling pathways, which often reside in specialized cell compartments. Photoreceptor cells for example have a ciliary outer segment that harbors specific proteins of the phototransduction cascade. The high sensitivity and precise performance of photoreceptor cells relies on its very specialized function, which seems to exclude the parallel existence of multisensory processes in the same cell type. However, recent findings support the hypothesis that magnetoreception of night-migratory songbirds is processed in the bird retina (see section Introduction). Furthermore, the putative magnetoreceptor Cry4a is expressed in European robin cone outer segments (Günther et al., 2018) and interacts with phototransduction proteins, in particular with European robin Gtα and LWO (Wu et al., 2020; Görtemaker et al., 2022). Such a non-canonical G protein mediated pathway needs to be compared with the classical coupling of European robin Gtα and LWO, but these interactions have not been studied so far. In the present work, we identified the second loop (LWO2) of the cytoplasmic loops in LWO as the main interacting region with Gtα/Giα. Previous studies on bovine rhodopsin using light scattering and peptide interference (König et al., 1989) or amino acid deletion and replacement experiments (Franke et al., 1992) concluded that the second and third loop represent the main binding sites required for functional interaction with the G protein. Earlier work using purified red-sensitive visual pigment (iodopsin) from chicken showed that the binding domains in rhodopsin and iodopsin for transducin are highly similar (Fukada et al., 1989). Furthermore, cone transducin when replaced for rod transducin in transgenic mice rod cells is a suitable substitute for the rod isoform (Mao et al., 2013). Collectively, these investigations indicated that transducin/rhodopsin or transducin/cone opsin interaction is similar in different species.

Truncation of Gtα/Giα at the C-terminus by three or six amino acids did not abolish binding to the LWO2 peptide, but the decrease of affinity in the case of Gt-6AA indicated the importance of the C-terminus in Gtα/Giα for LWO2 interaction. Previous studies showed that an 11-amino acid long peptide derived from the C-terminus of Gtα forms a complex with bovine opsin by causing an outward tilt of transmembrane helix 6, a pairing of helices 5 and 6, and a restructuring of helix 7 and 8 (Scheerer et al., 2008). A more recent cryo-electron microscopy study on human rhodopsin and an inhibitory Giα variant provided structural explanation of previous mutagenesis studies on the last 11 amino acids (Kang et al., 2018). The authors point to the contribution of the negative charge at the carboxyl group in F354 and the hydrophobic side chains in L353 and L348 for interaction with helix 8 and the hydrophobic pocket formed by transmembrane helices 3, 5, 6, and 7, respectively (Kang et al., 2018). Both of our truncated Gtα/Giα variants lack the C-terminal F and the Gt-6AA lacks also L353, but the binding process was not or only slightly disturbed. An explanation for this apparent inconsistency might be that our experimental design differs from structural studies mentioned above. The LWO peptides represent the soluble cytoplasmic part of the LWO that do not participate in the formation of the hydrophobic pocket. However, Kang et al. (2018) reported that the N-terminal α-helix in Giα provides a further interface for interaction with the second cytoplasmic loop in rhodopsin, which is in good agreement with our result identifying LWO2 as specific interacting surface.

We did not detect any interaction of peptide LWO3 (third cytoplasmic loop of European robin LWO) with Gtα/Giα. This could hint to a species difference, but our experimental setup using single peptide injection does not allow such a conclusion. So far, we could not employ the whole intact LWO protein that might involve the third cytoplasmic loop as well. However, we suggest that the critical interaction site in illuminated LWO is located on the second loop, for which we observed a high affinity binding process with a *K*_D_ value of 21.4 nM (Figure 3). Similar high-affinity binding was reported previously for light-activated bovine rhodopsin and purified native transducin (Salamon et al., 1996; Alves et al., 2005; Komolov et al., 2006; Ernst et al., 2007; Dell’Orco and Koch, 2011). These studies are in agreement with our results obtained for European robin specific Gtα/Giα and LWO peptides. Therefore, our data provides a kinetic framework for comparing binding processes in mammalian and bird photoreceptors.

Is the parallel existence of two primary sensory processes, phototransduction and magnetoreception, reasonable? One hypothesis suggests that Gtα/Giα and Cry4a form a complex that is part of a magnetoreceptive signaling pathway (Wu et al., 2020; Görtemaker et al., 2022). Such complex formation imposes a conceptual problem, if illuminated cone opsin activates the G protein in a high affinity binding process. Görtemaker et al. (2022) reported a *K*_D_-value of 35 nM for the interaction of non-myristoylated Gtα/Giα with Cry4a, which is close to our result with *K*_D_ = 21.4 nM, but not one or two orders of magnitude higher that would be necessary for an effective competition. Thus, under illumination, Cry4a could hardly compete with cone opsin (LWO), because Gtα is an abundant protein in photoreceptor cells and there is no evidence at the moment that Cry4a is expressed in high amounts. However, the situation relevant for magnetoreception is different. Blue-light photoexcitation of flavin containing Cry4a at 450 nm leads to a magnetically sensitive radical pair formation (Xu et al., 2021). Cry4a is expressed in long-wavelength sensitive single and double cones harboring LWO pigments (Günther et al., 2018). Avian LWO pigments have an absorbance maximum between 559 and 571 nm (Yokoyama et al., 2000). Taking the absorbance spectrum of zebra finch LWO with a maximum at 560 nm as a reference from the literature, shorter wavelengths at 450 nm would excite LWO only to less than 1% of its maximum [see Figure 4 in Yokoyama et al. (2000)]. This could still trigger activation of Gtα, but so far a comparative analysis of the photobiology of European robin Cry4a and LWO and their photo-excitation mediated interaction modes are missing to allow a definitive conclusion. However, another aspect of how birds can separate sensory information coming from phototransduction and magnetoreception is equally relevant for this topic. Worster et al. (2017) investigated in a theoretical study how light-sensitive magnetoreceptive molecules must be aligned to detect a weak magnetic field in the presence of changing ambient light intensities. Very recently, Chetverikova et al. (2022) provided experimental evidence for a regular orientation of double cones in the retina of European robin. The highly ordered double cone array would allow a separate processing of magnetic field information as predicted by Worster et al. (2017), when Cry4a is oriented and aligned in a fixed manner in double cones.

**Figure 6:**
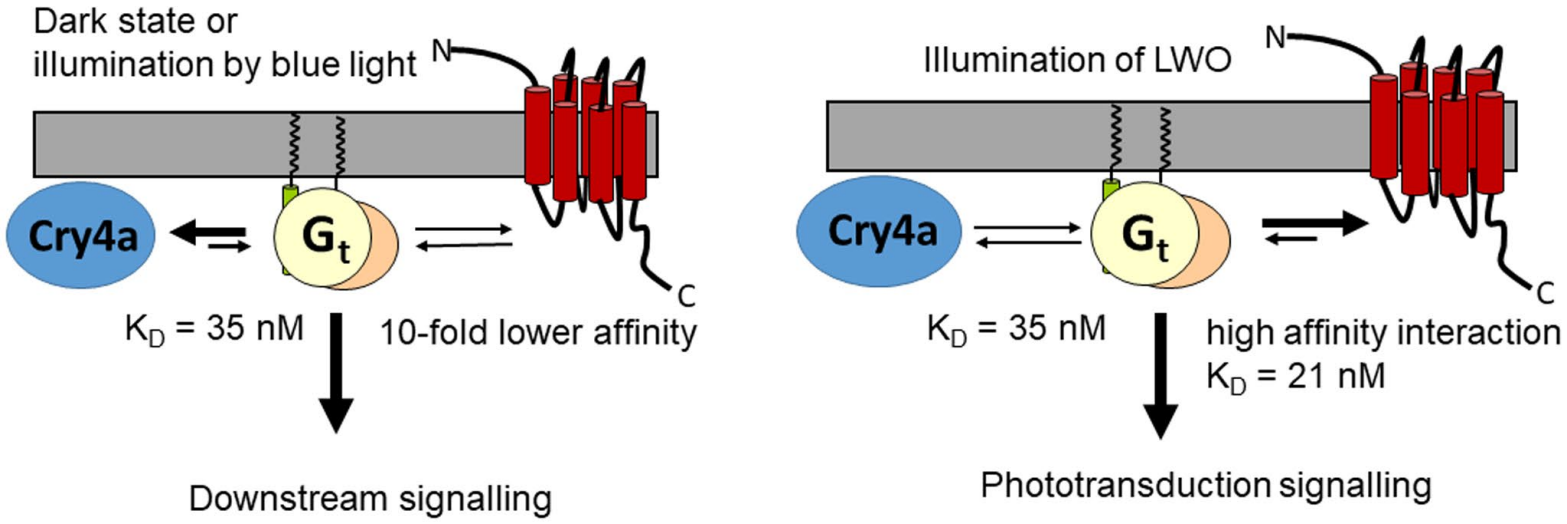
Hypothetical interaction scheme showing two scenarios, interaction of Gt with Cry4a, when LWO is not illuminated, and Gt interacting with LWO under illumination of LWO. The *K*_D_ value of 35 nM is taken from Görtemaker et al. (2022), *K*_D_ = 21 refers to the present work.

But can Gtα bind to LWO in darkness, thereby making any interaction with Cry4a unlikely? At present, we have no information about Gtα binding to dark adapted LWO, but evidence from the bovine Gtα/rhodopsin system exists. A supramolecular organization in disk membranes has been observed including a pre-assembled complex of Gtα and non-illuminated rhodopsin (Fanelli and Dell’Orco, 2005; Dell’Orco and Koch, 2011; Gunkel et al., 2015; Whited and Park, 2015). A SPR study reported a *K*_D_ of 360 nM for the binding of Gtα to dark-adapted rhodopsin (Dell’Orco and Koch, 2011), which is one order of magnitude lower than binding of Gtα/Giα to LWO2 (this study) or to Cry4a (Görtemaker et al., 2022). If we assume similar (moderate) affinities for the interaction of Gtα with non-excited LWO, we hypothesize that the binding of Gtα to Cry4a competes with LWO leading to downstream signaling relevant for magnetoreception (Figure 6). On the other hand, photoexcitation of LWO will favor its binding to Gtα and therefore trigger phototransduction.

## Data availability statement

The original contributions presented in the study are included in the article/Supplementary material, further inquiries can be directed to the corresponding author.

## Author contributions

CY, KG, and K-WK designed the study. CY, KG, and RW performed the experiments. CY, KG, RW, and K-WK analyzed the data. K-WK wrote the first draft of the manuscript. CY and KG contributed to writing of the manuscript. All authors corrected and approved the final version of the manuscript.

## Funding

This work was supported by two grants from the Deutsche Forschungsgemeinschaft to K-WK (SFB1372, Sig04 and GRK 1885/2).

## Conflict of interest

The authors declare that the research was conducted in the absence of any commercial or financial relationships that could be construed as a potential conflict of interest.

## Publisher’s note

All claims expressed in this article are solely those of the authors and do not necessarily represent those of their affiliated organizations, or those of the publisher, the editors and the reviewers. Any product that may be evaluated in this article, or claim that may be made by its manufacturer, is not guaranteed or endorsed by the publisher.
