# Supplementary material for: Kinetics of cone specific G-protein signaling in avian photoreceptor cells
Source: Front Mol Neurosci. 2023 Jan 17;16:1107025. doi: 10.3389/fnmol.2023.1107025 (PMC9887155; doi:10.3389/fnmol.2023.1107025)
Supplement: Supplementary file 1 [file Data_Sheet_1.PDF]

Supplementary Material

Kinetics of cone specific G-protein signalling in avian photoreceptor cells

Chad Yee[1,*], Katharina Görtemaker[1,*], Rieke Wellpott[1], and Karl-Wilhelm Koch[1,2,#]

[1]Department of Neuroscience, Division of Biochemistry, University of Oldenburg, D-26111 Oldenburg, Germany; [2]Research Center for Neurosensory Sciences, University of Oldenburg, 26111 Oldenburg, Germany.

* These authors contributed equally to the work

# to whom correspondence should be addressed:

[1]Department of Neuroscience, Division of Biochemistry, University of Oldenburg, 26111 Oldenburg, Germany; Tel: +49 0441 798 3640; e-mail: karl.w.koch@uni-oldenburg.de

Table S1
Primer sequences for cloning of truncated Gtα/Giα chimera.

	Primers	
	Sequence (5' to 3')	
1	tgaattctcgccagggttttc	Stop codon + Vector
2	gcagtccttgaggttctctttg	Minus 3 AA
3	gaggttctctttgatgatcacgtc	Minus 6 AA

Figure S1

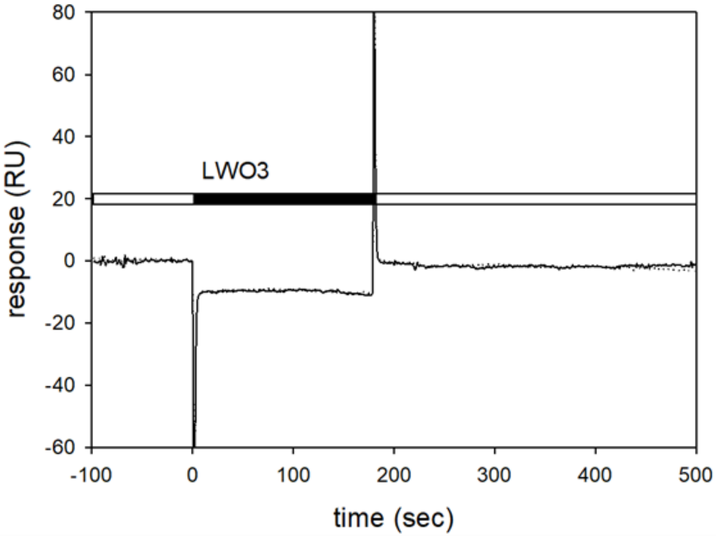

Figure S1. Sensorgram showing the injection of 200 nM LWO3 over a sensor chip surface coated with Gtα/Giα chimera. The black bar indicates the injection of the peptide, white bars show flowing of running buffer.

Figure S2

A

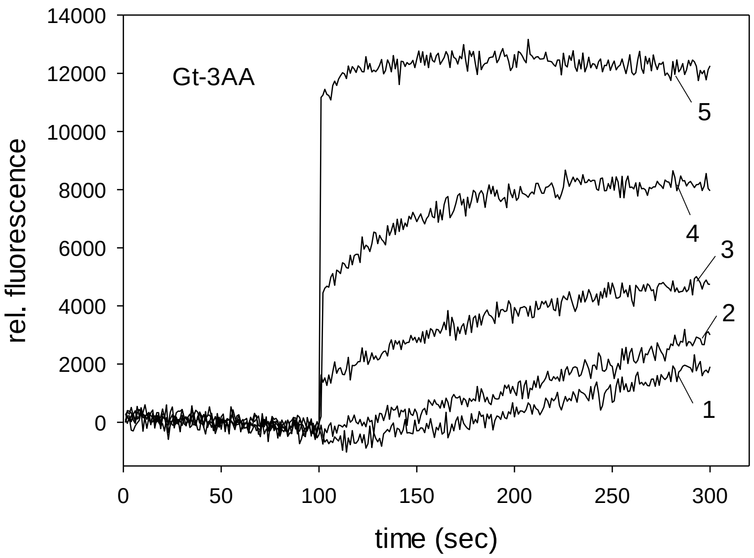

B

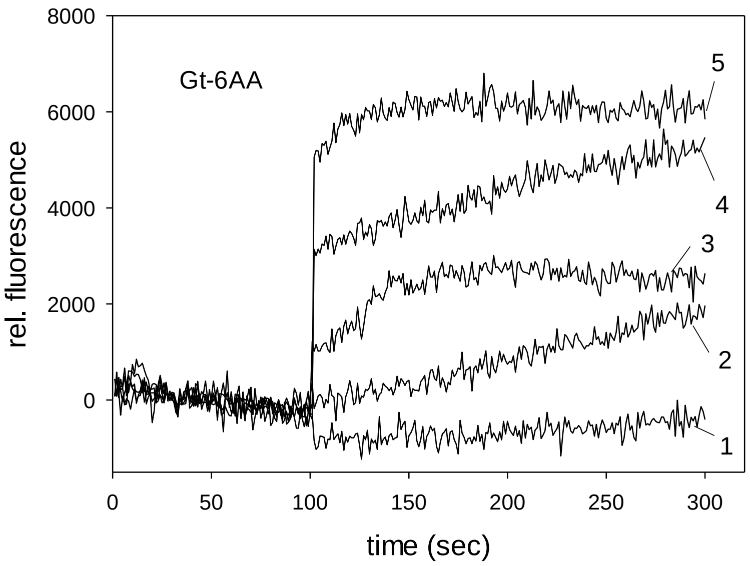

Figure S2. Functional test of the truncated Gtα/Giα chimera. **(A)** Recordings with truncated Gtα/Giα indicated as Gt-3AA. **(B)** Recordings with truncated Gtα/Giα indicated as Gt-6AA. Both Gtα/Giα were present at 1 μM in fluorescence buffer (50 mM Tris pH 7.4, 50 mM NaCl, 10 μM GDP). Trp fluorescence emission recording after injection of 50 μM AlF4-. Different $MgCl_2$ concentrations were present in fluorescence buffer as indicated and resulted in a successive increase of relative fluorescence emission: (1) 0.05 mM; (2) 0.1 mM; (3) 0.5 mM; (4) 1 mM; (5) 2 mM.

Figure S3

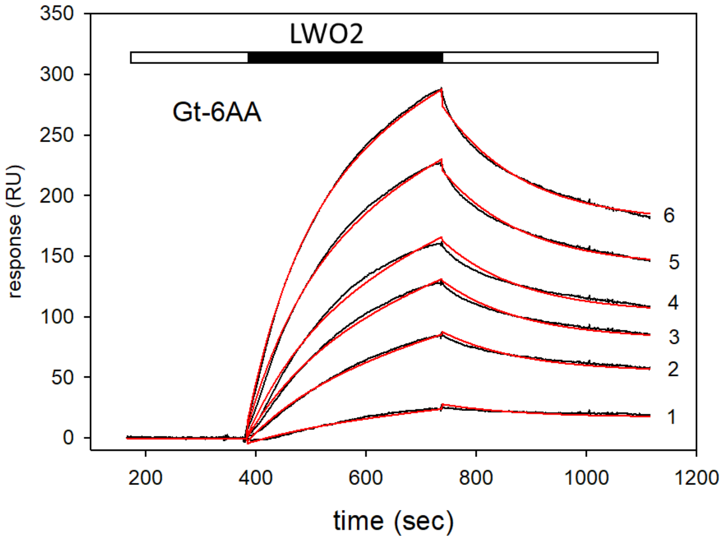

Figure S3. SPR recordings LWO2 injected over a Gt-6AA coated surface. Increasing concentration so LWO2 (black bar) are 100 nM (1), 250 nM (2), 374 nM (3), 500 nM (4), 750 nM (5), and 1000 nM (6). Global curve fitting (red lines) was performed using the two-state reaction model A+B ↔ AB ↔ AB* considering a conformational change. Fitting results are $k_{a1} = 4.65 \times 10^3\ M^{-1}\ s^{-1}$ and $k_{d1} = 4.34 \times 10^{-3}\ s^{-1}$, K_D = 933 nM.
